# Smoking and alcohol cessation intervention in relation to radical cystectomy: a qualitative study of cancer patients’ experiences

**DOI:** 10.1186/s12885-017-3792-5

**Published:** 2017-11-25

**Authors:** Susanne Vahr Lauridsen, Thordis Thomsen, Gudrun Kaldan, Line Noes Lydom, Hanne Tønnesen

**Affiliations:** 1 Department of Urology 2112, Inge Lehmanns Vej 7, Copenhagen University Hospital, Rigshospitalet, 2100 Copenhagen, Denmark; 2 Abdominal Centre, Copenhagen University Hospital, Rigshospitalet, Copenhagen, Denmark; 3 University of Copenhagen, Health & Medical Sciences, Copenhagen, Denmark; 4 Clinical Health Promotion Centre, Bispebjerg & Frederiksberg Hospital, Copenhagen University Hospitals, Copenhagen, Denmark; 5 Clinical Health Promotion Centre, Health Sciences, Lund University, Lund, Sweden; 6 Health Science, University of Southern Denmark, Odense, Denmark

**Keywords:** Smoking cessation, Alcohol cessation, Surgery, Qualitative study, Bladder cancer, Cancer patient

## Abstract

**Background:**

Despite smoking and risky alcohol drinking being modifiable risk factors for cancer as well as postoperative complications, perioperative cessation counselling is often ignored. Little is known about how cancer patients experience smoking and alcohol interventions in relation to surgery. Therefore the aim of this study was to explore how bladder cancer patients experience a perioperative smoking and alcohol cessation intervention in relation to radical cystectomy.

**Methods:**

A qualitative study was conducted in two urology out-patient clinics. We conducted semi-structured in-depth interviews with 11 purposively sampled persons who had received the smoking and alcohol cessation intervention. The analysis followed the steps contained in the thematic network analysis.

**Results:**

Two global themes emerged: “smoking and alcohol cessation was experienced as an integral part of bladder cancer surgery” and “returning to everyday life was a barrier for continued smoking cessation/alcohol reduction”. Participants described that during hospitalization their focus shifted to the operation and they did not experience craving to smoke or drink alcohol. Concurrent with improved well-being or experiencing stressful situations, the risk of relapse increased when returning to everyday life.

**Conclusions:**

The smoking and alcohol cessation intervention was well received by the participants. Cancer surgery served as a kind of refuge and was a useful cue for motivating patients to quit smoking and to reconsider the consequences of risky drinking. These results adds to the sparse evidence of what supports smoking and alcohol cessation in relation to bladder cancer patients undergoing major surgery and point to the need to educate healthcare professionals in offering smoking and alcohol cessation interventions in hospitals. The study also provides knowledge about the intervention in the STOP-OP study and will help guide the design of future smoking and alcohol cessation studies aimed at cancer patients undergoing surgery.

**Electronic supplementary material:**

The online version of this article (10.1186/s12885-017-3792-5) contains supplementary material, which is available to authorized users.

## Background

Radical cystectomy is a highly morbid procedure and complication rates vary from 30 to 64% [1, 2] regardless of operative technique [3]***.*** Both smoking and risky alcohol drinking are modifiable risk factors for cancer as well as postoperative complications [4–6]. In addition, smoking is the major and most modifiable risk factor for development of bladder cancer [7]. Continued smoking and risky alcohol drinking affect pathophysiological mechanisms, such as tissue perfusion and oxygen delivery, ciliary and immune function, surgical stress response, arrhythmias, and bleeding time [8–10], all of which are beneficial for postoperative recovery. Observational studies show that smoking cessation reduces the risk of recurrence and cancer related death [11] while continued smoking leads to decreased survival, decreased quality of life and increased risk of readmission within 30 days in bladder cancer patients [12–14]. Interventional studies show that intensive smoking and alcohol cessation intervention 6–8 weeks before elective surgery reduces the incidence of postoperative morbidity to about half [5, 6, 15] while a programme with one preoperative meeting showed no effect on surgical risk reduction for other groups of cancer patients undergoing surgery [5, 16]. Conclusive recommendations for the timing of smoking and alcohol cessation interventions are therefore still lacking. On one hand cancer diagnosis has been described as “a window of opportunity” to change lifestyle [17–19]; patients with cancer diagnosis are more likely to quit smoking than patients not diagnosed with cancer [20–22]. On the other hand, half of patients with cancer who smoked before diagnosis continue to smoke during treatment [13, 20, 23]. This could be attributed to the lack of effective interventions, but also intrusive cancer treatment programs may counteract intentions to quit, due to challenging changes in bodily function and body image [24, 25]. A commonly described barrier that may prevent health professionals from offering patients smoking cessation interventions is that addressing smoking will be unwanted by the patient or might imply judgement from the healthcare professionals [26, 27]. It is well-known that cancer survivors are interested in lifestyle-interventions [28], but still little is known about how cancer patients experience these interventions [29, 30]. To our knowledge no studies have investigated cancer patients’ experiences of following an intensive smoking and alcohol cessation intervention in relation to major surgery. Therefore, the aim of this study was to explore how bladder cancer patients experienced a perioperative smoking and alcohol cessation intervention in relation to radical cystectomy.

## Methods

The protocol for this study was written in compliance with the COREQ guidelines [31]. The Danish Scientific Ethical Committee System (16040244) evaluated the study protocol and found formal appraisal of the study to be unnecessary. The Danish Data Protection Agency (2012–58-0004) approved the study. All patients received both oral and written information and signed informed consent before interviewing.

A qualitative descriptive design was adopted to obtain in-depth knowledge about how newly diagnosed cancer patients experienced the intensive smoking and alcohol cessation interventions in relation to major bladder surgery. Inspired by a phenomenological – hermeneutical method [32] we conducted semi-structured in-depth interviews. This study design provides the possibility to understand the impact of the smoking and alcohol cessation intervention in relation to radical cystectomy from the patient perspective. The study formed part of the STOP-OP study (Stop smoking and alcohol drinking before operation for bladder cancer), a randomized controlled trial evaluating the effect on postoperative complications and quit rates of an intensive smoking and alcohol cessation intervention in relation to radical cystectomy [33]. The STOP-OP study has enrolled patients since November 2014 and is expected to be finalized at the end of 2017.

### Sampling strategy

To elucidate the research question we used the principle of purposeful sampling with maximum variation [34] to achieve a varied sample with respect to: age, smoking, alcohol drinking or both smoking and alcohol drinking and success or failure in abstaining. Data was collected until saturation [35].

### Participants and setting

The interviews were conducted between November 2016 and May 2017. Fourteen patients were invited to participate in the qualitative study, three declined participation. All participants were recruited from the STOP-OP intervention group and asked for participation by the study nurse after completion of the 6-weeks intervention. Three patients had the interview conducted at 3 months follow-up, four at 6 months follow-up and four at 12 months follow-up.

The interviews lasted 20–40 min and were all conducted in two urological departments in connection with a scheduled out-patient visit. Participants were offered to have the interview in their own home, but everyone preferred to be interviewed in the outpatient clinic.

### The STOP-OP study

The STOP-OP study is an ongoing multi-center; randomized, controlled trial with patients allocated to either a smoking and/or alcohol cessation intervention initiated shortly before radical cystectomy and lasting for a total of 6 weeks; or to treatment as usual. Due to ongoing efforts to accelerate diagnosis and treatment of patients at suspicion of having cancer, in Denmark, the timeframe for intervening before cancer surgery is maximum 14 days, often less. Inclusion criteria for the STOP-OP study are: patients scheduled for radical cystectomy for bladder cancer and who smoke daily and/or drink at least 3 units of alcohol daily. One unit contains 12 g of ethanol.

### The smoking and alcohol cessation intervention

The intervention followed the principles of the Gold Standard Programme (GSP) [36] (Table 1 shows the content of the GSP). The principles of motivational interviewing, balanced decision making and the trans-theoretical model of change are the underlying tenets of the programme [37]. The participants received counselling sessions before and after surgery during a period of 6 weeks. Trained smoking and alcohol cessation counselors who, were also experienced urology nurses, provided the intervention.

**Table 1:** Gold Standard Programme for smoking and alcohol cessation intervention

Patient education programme	
First meeting (before admission)	
◦ Level of motivation, ambivalence, pros and cons	
Second meeting (after 1 week)	
◦ Dependence, withdrawal symptoms (experience and expectations)	
Third meeting (after 2 weeks)	
◦ Relapse (description and management)	
Fourth meeting (after 3 weeks)	
◦ Benefits of short and long term smoking and/or alcohol abstinence	
Fifth meeting (after 5 weeks)	
◦ Continued smoking abstinence and/or reduced alcohol intake following intervention	
At each meeting	
Smokers: Personalized Nicotine Replacement Therapy (NRT) in accordance with patient preferences and nicotine dependency	
Risky drinkers: Thiamine and B-vitamins (300 mg × 7 weekly)	
Alcohol withdrawal prophylaxis and treatment (chlordiazepoxide 10 mg as required)	
Disulfiram (200 mg × 2 weekly) supervised at weekly meetings (not administrated if patients test positive on breath test)	
All: Hemoglobin, liver enzymes and alcohol biomarkers (blood, urine), CO and alcohol breath test, lung function test	
The study medication is provided for free and transportation for the weekly meetings will be reimbursed. Patients have telephone access to the research nurse	

### Data collection and analysis

Data was collected using face-to-face interviews which were undertaken by two research nurses (TT and GK) who were not directly involved in the STOP-OP intervention or familiar with the patients. A semi-structured interview guide with open-ended questions was prepared beforehand to ensure coverage of the research question. The interviewer asked broad questions to explore the individual person’s experience (See Additional file 1). The introductory question was *“How did you experience having to deal with the cancer surgery and quitting smoking/drinking at the same time?”*

**Fig. 1:**
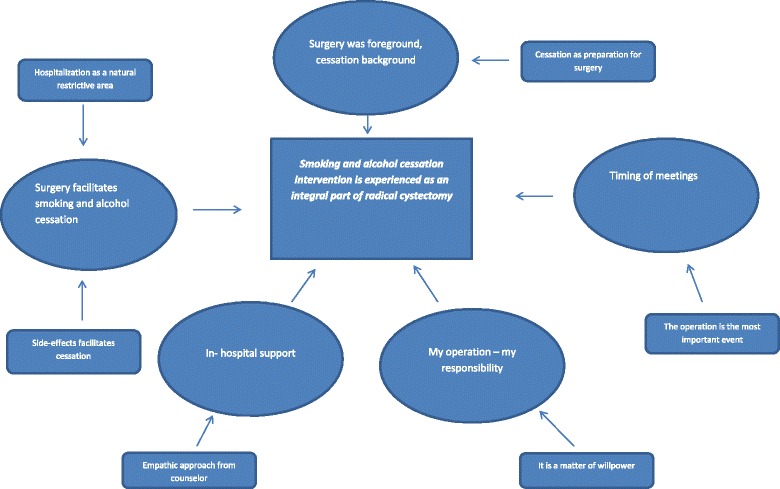
Thematic network for the main finding: the smoking and alcohol cessation intervention is experienced as an integral part of bladder cancer surgery

All interviews were recorded digitally and transcribed verbatim. Interview transcripts were managed using the software program QSR NVivo (Version 11). Coded data were used to generate potential themes to facilitate analysis. The analysis followed the steps contained in the thematic network analysis [38]. Themes were identified and the network constructed, (Fig. 1 shows the network for the main finding). In the analysis process we kept on exploring the network by consulting the interview text and finally we interpreted our findings.

### Trustworthiness.

We assessed the rigor of our research regarding credibility, dependability, transferability, and confirmability [35, 39] according to the description of Guba [40]. To establish credibility we chose participants from the three different interventions (smoking, alcohol drinking or both smoking and alcohol drinking). During the interviews, experiences described by the participants were restated by the interviewers who then asked the participant to validate the statements. In addition the interviewers had no former contact with the participants or experience from bladder cancer treatment. Thus, the participants were encouraged to be frank from the outset of each session and they were assured that there was no right or wrong answers to the questions posed. Dependability was sought by step-wise replication as the analysis was undertaken by LNL and SVL who discussed their individual findings in each separate interview. Next the findings were discussed in the whole research group to identify the multiple realities experienced by the participants. The description of participant characteristics makes it possible for the reader to assess transferability to other bladder cancer patients. Confirmability was sought obtained through the structure of the thematic net-work identified and the quotations included in the presentation of the findings.

## Results

Eleven participants aged 43–77 were interviewed, 10 men and 1 woman, mean pack-year of smoking was 43, indicating that the participants were all heavy smokers (Table 2 lists characteristics of participants). At the time of the interview five participants were still smoke-free or had reduced alcohol drinking.

**Table 2:** Characteristics of the 11 participants

Age at diagnosis, *median (range)*	58 (43–77)
Age at smoking debut, *median (range)*	15 (7–19)
Pack years, *median (range)*	43 (26–54)
Fagerström Nicotine Dependency score, *median (range)*	4.5 (3–6)
Smoking cessation intervention (*n*)	6
Alcohol cessation intervention (*n*)	3
Smoking and alcohol cessation intervention (*n*)	2
*Smoke and alcohol free during the 6-weeks intervention* (*n*)	
Yes	8
No	3
*Smoke-free /alcohol reduction at the interview* (*n*)	
Yes	5
No	6
*Living with a smoker* (*n*)	
Yes	4
No	7
*Surgical procedure*(*n*)	
Ileal conduit	8
Neobladder	2
Continent cutaneous diversion	1

A global theme emerged to explain the participants’ experience of participating in a smoking and alcohol cessation intervention in relation to radical cystectomy. We present this as *Smoking and alcohol cessation was experienced as an integral part of bladder cancer surgery.* Another global theme captured the participants’ experience of the time after surgery; *Returning to everyday life was a barrier for continued smoking cessation/alcohol reduction.*

### Global theme: Smoking and alcohol cessation was experienced as an integral part of bladder cancer surgery

The operation was the most urgent issue for participants and the smoking-alcohol cessation programme merely a minor part. The participants did not perceive the smoking and alcohol intervention and undergoing surgery as two separate entities. Rather, the intervention was perceived to be an integral part of preparing for surgery. Moreover, in addition to being a smoking-alcohol cessation intervention, participants experienced that the intervention meetings provided them with an extra source of support in the bladder cancer pathway. The operation was the key issue in the participants’ lives, and therefore the timing of intervention meetings was of major importance for their motivation to participate. Participants did not find it difficult to stop smoking or drinking alcohol in relation to radical cystectomy, partly because smoking was prohibited in and around the hospital, partly because they experienced side-effects from surgery. Several participants reflected upon their personal responsibility both towards own health, family and also towards the smoking and alcohol cessation counsellor and society.

### Surgery was foreground, cessation background

Participants described that the cancer diagnosis and undergoing radical cystectomy was a stressful situation, but despite this participants were willing to engage in the programme. Participants did not see the smoking and alcohol intervention programme and undergoing surgery as two separate entities. Rather the intervention was seen as part of the preparation for surgery.

*Well I haven’t seen it as though I had to do two things* (P10, alcohol intervention)

*Well, for me the cancer was the major issue and the other stuff sort of minor, right* (P2, smoking intervention)

*It (the smoking intervention) didn’t become more of a main issue because I had to have the surgery and it wasn’t harder because I had to quit smoking and have the surgery at the same time sort of* (P3, smoking intervention)

### Timing of meetings

As the operation was the key issue in the participants’ lives, timing of meetings in the programme was of importance. All meetings were planned in connection to scheduled out-patient visits or as telephone calls. This was of great importance to the participants because many had a long way to travel to the hospital.

The participants stated that the intervention meetings were supportive; they made them feel more comfortable and allowed them to also ask questions related to their treatment in general.

*I said that the meetings had to be when I was in here (the hospital), right? If they could do that, then fine* (P4, smoking intervention)

*And I felt incredibly comfortable with it all. Especially knowing that I was part of a process and part of a programme* (P11, alcohol intervention)

### Surgery facilitates smoking and alcohol cessation

Participants did not find it difficult to stop smoking or drinking alcohol in relation to radical cystectomy. The restrictive situation of being inside the hospital where smoking was prohibited was a help to adhere to smoking cessation. Side-effects of surgery such as nausea, oral thrush or changes in taste due to medication also meant that participants felt no desire to smoke or drink alcohol during the hospital stay. Participants felt it was “strange” that “I did not need it during hospitalization” when they described the ease of abstinence at this time. Participants in the alcohol intervention group were not surprised that abstinence was easy, because they did not feel addicted to alcohol beforehand. It seemed that surgery served as a kind of refuge where the combination of the acceptance of hospitals being smoke-free areas and the side-effects of abdominal surgery facilitated smoking cessation.

*When I go through that door downstairs, well then I can’t smoke. Because – well I can’t because it’s not allowed. So I don’t* (P1, smoking and alcohol intervention)

*It was actually as though you have to now because you’re going through all this and, strangely, I didn’t miss it* (P7, smoking intervention)

*I got a yeast infection in my mouth and everything tasted like kerosene or diesel, right. I couldn’t eat anything and I lost about 6-7 kilos while I was there. And I didn’t even think about smoking at all* (P8, smoking intervention)

### My operation – My responsibility

Even though most participants felt that cessation was a personal responsibility and a matter of willpower, they also described the motivation to participate in the intervention as an obligation either towards their own health, family, healthcare professionals, or society. Most participants were aware of the importance of their behavior and the patient-counsellor interaction. The wish to be “a good patient persona” doing what both family and healthcare professionals expected encouraged the participants to cessation.

*Now, at last, someone offered to help me and it’s a good offer of help with nicotine replacement therapy and all that… Yeah, you don’t want to lose face and have to say… And because my family, we’ve talked about the cancer in my family and ah also that you have to stop smoking and all that, you know* (P2, smoking intervention).

Repeatedly participants expressed that people are personally responsible and that cessation can only be achieved through willpower. One participant also reflected upon the consequences of the hospital not requiring patients to stop smoking and drinking alcohol in relation to surgery.

*I ought to join a quit programme of my own accord at home, right…. For me it’s mainly about willpower, I mean I lack willpower. I don’t have the last bit* (P7, smoking intervention)

*You should more or less demand that people stop smoking before major surgery; I mean surgery is expensive for society. And I think it would be reasonable, as part of the treatment, to ask people to be abstinent* (P11, alcohol intervention)

### In-hospital support

The empathic approach underlying the intervention was of importance and was seen both as a support to cessation and as a support in the postoperative period. Several participants felt more motivated when healthcare professionals encouraged them to cessation. Some underpinned that a paternalistic approach would have made them continue smoking out of spite.

*For me it’s positive that she’s such a nice person. She doesn’t say you can’t do this or you’re not allowed that because that would make me become stubborn and say I’m going to smoke regardless (laughing)* (P9, smoking intervention)

*I got raised fingers from doctors and nurses that it would be a good idea if I didn’t smoke in connection with my surgery* (P2, smoking intervention)

### Global theme: Returning to everyday life was a barrier for continued smoking cessation/alcohol reduction

Returning to everyday life and feeling better played an important role in the risk of relapse. Context specific factors like negative affect, social-food situations and habit-driven smoking and drinking were given as reasons for relapse.

### Lifestyle

Restoration to health and resumption of one’s habitual lifestyle were described as critical for either maintaining lifestyle changes or for relapse. Participants expressed that feeling better or stressful situations were reasons for relapses. Despite the fact that only one participant in the alcohol intervention group thought he was a heavy drinker, all participants described that they maintained changes in lifestyle after completion of the intervention. These changes were motivated by referring to the benefits of weight loss, improved sleep and being more conscious of listening to their body. No participants mentioned heavy alcohol drinking as the motivation for lifestyle changes.

*Maybe I just felt a little better, I don’t know, and then when I was told that the cancer had possibly spread I thought sod it all – I’m going to have a fag. And then – well before you know it you’re smoking as much as you used to* (P3, smoking intervention)

*But then I started to feel really, in fact really normal again. So I started to smoke because I was able to start working more, right* (P8, smoking intervention)

*I found out that I’ve lost almost 10 kilos …. So there’s no reason to start drinking and putting on weight again* (P7, alcohol intervention)

For some participants smoking and drinking alcohol were major parts of socializing with family and friends and this was a barrier to maintaining the new lifestyle. Some mentioned that the chance of maintaining cessation would have been increased by attending group sessions as they would then become part of a new social group.

*Yes… It happened--- I think it was at a party or something I was talking to a family member and well – okay I’ll just smoke one cigarette with you, right. And then I started again* (P7, smoking intervention)

*It could be a Sunday lunch with the family; it could be with close friends, especially if you’re on holiday with friends, right. It’s nice with some rose wine and a barbecue… I haven’t cut down on situations but I’ve cut down on how much I drink in those situations* (P10, alcohol intervention)

### Breaking habits

The replacement of a habitual response with an alternative response in situations where the participants used to smoke or drink alcohol could overrule habits and for some this was a deliberate coping strategy.

*As soon as I’ve finished eating and I can feel the craving coming, I get up and take the plates out and start washing them or whatever I can think of* (P4, smoking intervention)

*Yes, I thought a lot about that, it was hard to find a replacement. When I’m out I always bring sparkling water. In that way I fill myself with something else* (P10, alcohol intervention)

Some participants experienced family as supportive, but the converse was also seen. Patients who experienced pressure from relatives to stop smoking emphasized that the decision was theirs alone and they experienced the family’s concern as “nagging” rather than caring.

*It’s both that you can motivate each other because my wife, I mean she can motivate me but actually she can also demotivate me. She gets upset and also a bit mad at me when I keep on smoking. I can easily understand her reaction* (P7, smoking intervention)

*Particularly when my wife sees the ashtray she tells me off (…). Yes if someone starts saying that I have to, then I won’t, not for anything* (P9, smoking intervention)

Replacing a habitual response with an alternative response was successful for some to maintain cessation and to reduce consumption. Others were very confident that they would not relapse.

*I don’t think so. I think I would have by now… already have had withdrawal symptoms and now I’m convinced that nothing will happen. So I’m quite sure that I’m over it. And I can thank the smoking cessation counselors for that* (P2, smoking intervention)

This man was interviewed at 12 – months follow-up and was still smoke-free.

## Discussion

The participants interviewed in this study experienced the smoking and alcohol intervention as a relevant offer in relation to major bladder cancer surgery. The intervention and surgery were the main factors supporting cessation; both the timing and the setting were pointed out as important and the empathic approach inherent in the intervention and the contact to the counselors was also experienced as supportive. Despite the fact that cessation during hospitalization felt easy for all participants, returning to everyday life challenged continued smoking abstinence and reduced alcohol intake in most participants.

There are some limitations to this study. Participants were all recruited from the intervention group in the STOP-OP study and are likely to represent a somewhat motivated group, having voluntarily taken part in the STOP-OP trial. Furthermore only one woman was interviewed. As bladder cancer is four times more likely to occur in men [41], we found this distribution of gender acceptable.

A strength of this study is that participants were interviewed at different times after surgery and thus provided knowledge about the impact of the 6-week intervention both short-term and long-term.

The meetings in this study were all held at the urological department, often in connection with bladder cancer follow-up visits and the counselors were all experienced urology nurses. This might have contributed to the experience of the smoking and alcohol cessation intervention as an integral part of surgery. This is in line with the study from Sharp et al. who also found that integrating the smoking cessation process with other care needs, and viewing the intervention as an intrinsic part of the patient’s anti-cancer treatment, facilitated smoking cessation [42]. The participants’ experience of the smoke-free hospital as supportive of cessation confirms the effect of the WHO recommendation that all people should be protected from exposure to tobacco smoke and that all indoor workplaces and indoor public places should be smoke free. [43].

It is interesting that several studies have identified that the attitudes of healthcare professionals are a major barrier to offering smoking cessation interventions [26, 27, 44, 45], while studies exploring both cancer and non-cancer patients’ attitudes show the opposite [29, 30, 46, 47]. This contradiction in attitudes can be overcome by communicating the patients’ perspectives on clinical health promotion to healthcare professionals.

This study adds to the evidence that every contact with a cancer patient poses an opportunity for healthcare professionals to engage in health promotion.

Laying the blame for continued smoking on themselves and the belittlement of nicotine dependency among the study participants is consistent with the findings of Morphett et al.[48]. They also found that despite meticulous explanations about the neurobiological basis of addiction the participants stated they remained personally responsible for continued smoking. This might explain why returning to everyday life is a challenge. As patients recover from surgery, they will increasingly encounter situations where they used to smoke or drink alcohol and subsequently, face the risk of succumbing to the power of usual habits.

Our findings point to the feasibility of enhancing the effect of physicians’ advice [49] about smoking and alcohol cessation by involving urology nurses educated in smoking and alcohol counselling in delivering cessation interventions in relation to surgery.

## Conclusion

The smoking and alcohol cessation intervention was well received by the participants. This is the first study exploring heavy smokers and risky drinkers’ experiences of a 6 week smoking and alcohol cessation intervention in relation to bladder cancer surgery. Our results add new insight to existing evidence that patients do want support to stop smoking and risky drinking during hospitalization, but also to the need for continued support when returning to everyday life. Our findings point both to the need to educate healthcare professionals in providing smoking and alcohol cessation interventions and to further explore if a follow-up intervention after the initial hospital-based 6 week intervention would support long-term cessation. If the results of the STOP-OP study support these findings we will look into the feasibility of addressing smoking cessation and alcohol reduction at each follow-up meeting in the urological outpatient clinic.

## Additional file

Additional file 1: Interview guide. The interview guide shows the five areas and the questions asked during the interview. (DOCX 19 kb)

## Abbreviations

GSP: Gold Standard Programme
STOP-OP: Stop smoking and alcohol drinking before operation for bladder cancer
