# Supplementary material for: Smoking and alcohol cessation intervention in relation to radical cystectomy: a qualitative study of cancer patients’ experiences
Source: BMC Cancer. 2017 Nov 25;17:793. doi: 10.1186/s12885-017-3792-5 (PMC5702236; doi:10.1186/s12885-017-3792-5)
Supplement: Additional file 1: — Interview guide. The interview guide shows the five areas and the questions asked during the interview. (DOCX 19 kb) [file 12885_2017_3792_MOESM1_ESM.docx]

| About the smoking and/or alcohol cessation intervention. | - What were your thoughts when you initially heard about the smoking and/or alcohol cessation intervention? - Describe what it was like to participate in the smoking and/or alcohol cessation intervention - What was it like for you to have to consider both having major surgery for bladder cancer and quitting smoking/risky drinking - What was supportive for you in the smoking and/or alcohol cessation intervention - Do you have any suggestions for improvements – anything that could support you more or make abstinence easier in relation to surgery |
| --- | --- |
| Perspectives on motivating aspects of the GSP | - **If the participant was abstinent before surgery** - Describe a situation that motivated you to quit before surgery |
|  | - **If the participant was not abstinent before surgery** - Have you experienced any situations after surgery that motivated you to quit or stay abstinent? - Who supported you in quitting? - How did you experience their support? |
| Perspectives on barriers in the GSP | - Describe a situation that made it difficult for you to quit before surgery - Describe a situation that made it difficult for you to quit after surgery |
| Perspectives on physical benefits/disadvantages of smoking/alcohol cessation | - Do you feel any physical changes after you quit smoking/drinking? What changes (NB! Can the changes be distinguished from changes caused by the surgery) How do you think your postoperative recovery would have been, if you had not participated in the smoking/alcohol cessation intervention |
| Perspectives on advantages/disadvantages of smoking/alcohol cessation | - What has your mood been like after quitting? - How do you think your mood would have been, if you had not participated in the smoking/alcohol cessation intervention |
| End of the interview | - Do you have anything to add ? |
